# App-technology to improve lifestyle behaviors among working adults - the Health Integrator study, a randomized controlled trial

**DOI:** 10.1186/s12889-019-6595-6

**Published:** 2019-03-07

**Authors:** Stephanie E. Bonn, Marie Löf, Claes-Göran Östenson, Ylva Trolle Lagerros

**Affiliations:** 1 Clinical Epidemiology Division, Department of Medicine (Solna), Karolinska Institutet, Eugeniahemmet T2, 171 76 Stockholm, Sweden; 2 Department of Biosciences and Nutrition, Karolinska Institutet, Stockholm, Sweden; 3 Department of Medical and Health Sciences, Linköping University, Linköping, Sweden; 4 Department of Molecular Medicine and Surgery, Endocrine and Diabetes Unit, Karolinska Institutet, Karolinska University Hospital, Stockholm, Sweden; 5 Clinic of Endocrinology, Metabolism and Diabetes, Department of Medicine, Karolinska University Hospital Huddinge, Stockholm, Sweden

**Keywords:** Adults, Body composition, Exercise, HbA1c, Healthy lifestyle, Metabolic health, Mobile application, Quality of life, Randomized controlled trial, Smartphones

## Abstract

**Background:**

Mobile health, mHealth is recognized as a strategy to improve lifestyle behaviors. Research targeting specific lifestyle behaviors has shown that interventions using smartphones can be effective. However, few studies have evaluated solutions with multicomponent interventions, tailoring the intervention to the specific needs of the participant using a combination of mHealth and conventional treatment. To accomplish this, we developed Health Integrator, an mHealth platform with services and offers in the areas of diet, physical activity, sleeping habits, stress, alcohol and tobacco use. In the system, the user selects an area of intervention together with a health coach and set weekly goals. This study protocol presents the design and methodology of the Health Integrator Study, a randomized controlled trial to promote improved lifestyle behaviors.

**Methods:**

A three-arm parallel randomized controlled trial (1:1:1) is conducted in the Stockholm County, Sweden. In total, 209 employees at a four different companies representing both white and blue collar workers, have been recruited.

Participants are randomized to either a control group or to one of two intervention groups receiving a 3-month lifestyle behavior change program including either 1) use of Health Integrator and monthly health coaching sessions or 2) only Health Integrator.

At baseline and follow-up after 3- and 6-months, all participants answer questionnaires assessing lifestyle behaviors and quality of life. At baseline and the 3-month follow-up (end of intervention period), weight, height, waist circumference and blood pressure are measured, and all participants wear an Actigraph accelerometer for 7 days to assess physical activity. Blood lipid profile and HbA1c are measured among all participants at baseline. If baseline measures fall outside the normal range, a second measurement is done after 3 months.

**Discussion:**

The Health Integrator Intervention Study will evaluate if a personalized intervention combining mHealth and conventional programs for lifestyle change, with or without additional health coach sessions, can improve lifestyle behaviors and quality of life. Based on the results from this trial, Health Integrator can easily be implemented within a broad public.

**Trial registration:**

ClinicalTrials.gov Identifier: NCT03579342. Retrospectively registered, first submitted May 8, 2018.

## Background

Lifestyle is the single most important factor to improve health and decrease premature death [1]. There has been an exponential development of preventive initiatives using digital solutions for implementing lifestyle change, and tracking different types of health data such as exercise, diet, medications, stress and more. mHealth, defined by the World Health Organization, WHO, as “medical and public health practice supported by mobile devices, such as mobile phones, patient monitoring devices, personal digital assistants and other wireless devices” [2], has grown rapidly over the last couple of years. Previous mHealth studies have proven that it is possible to conduct lifestyle interventions using smartphones [3]. If the aim is better health at a lower cost, previous studies can be seen as proof-of-concept. Studies evaluating systems that are developed for dissemination in the population are crucial.

Since smartphones entered the market about a decade ago, they have become an important part of everyday life and can be used for tracking health and health related behaviors. In Sweden, 90% of the population own a smartphone and 98% have access to internet at home; ownership and usage is independent of socioeconomic status [4]. With this in mind, interventions using internet and smartphones may bridge the gap between individuals’ need for personalized health interventions and the regular health care’s lack of capacity to support these individuals. Additionally, the WHO Global Plan of Action on Workers’ Health clearly states that the health of workers must not only be protected, but also promoted in the workplace [5]. Intervening in work places may be a challenge due to different work tasks and schedules, but tailored lifestyle interventions using mHealth may be a solution.

Based on evidence from previous research, we have built a new digital platform for lifestyle change, called Health Integrator, to be used for example in work place settings. The platform is the product of a 5 day intense “design sprint” process, involving individuals from science, business, and innovation. It was further developed and prototyped through service design in collaboration with relevant stakeholders before it was launched to study participants in the described randomized controlled trial.

The Health Integrator platform offers a variety of public, private and community services for behavior change in different domains such as smoking, alcohol, physical activity, diet, stress, and sleep. This trial is a scientific evaluation of the efficacy of Health Integrator with an assortment of different lifestyle interventions to personalize health. The Health Integrator system is funded by the European Institute of Innovation and Technology, EIT, a European initiative to empower innovators to create innovative solutions for health as a part of Horizon 2020.

To scientifically evaluate the Health Integrator system, we are now conducting a randomized, controlled trial to determine the effectiveness of lifestyle intervention using mHealth.

### Aim

The aim of this paper is to describe the study design and methodology of the Health Integrator Intervention. We aim to evaluate if a digital platform accessible via a smartphone-app offering lifestyle intervention, with and without additional health coach guidance, can be used to make lifestyle changes and improve health related quality of life (primary outcome) in gainfully employed persons. Secondary outcomes include improving levels of blood pressure, body composition, blood lipids, HbA1c and other health related lifestyle factors.

### Hypothesis

We hypothesize that the subjects assigned to active intervention will have improved health related quality of life as well as improved cardiovascular risk factors after 3 months compared to the control group, and that improvements will last another 3 months after the intervention has ended. We further hypothesize that subjects in the intervention group receiving in-person support will achieve greater improvements than those with only digital support.

## Methods/design

### Study design

**Fig. 1:**
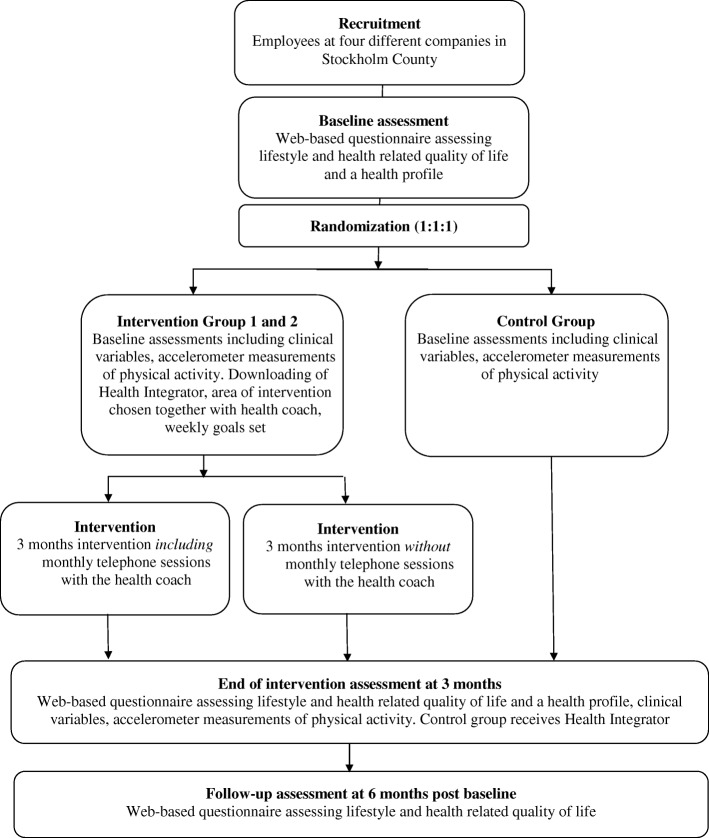
Flow-chart of the Health Integrator study design

The Health Integrator intervention study is a three-arm parallel randomized controlled trial (1:1:1) conducted in the Stockholm County of Sweden. Assessments are done at baseline, and post intervention at follow-up after 3 and 6 months. See Fig. 1. The study protocol follows the SPIRIT 2013 Statement [6, 7] and the intervention is described according to the CONSORT EHEALTH checklist [8].

### Inclusion and exclusion criteria

Inclusion criteria are: age above 18 years, being able to read and understand Swedish well enough to understand the study aims and informed consent, having access to and being able to use a smartphone, and giving informed consent to participate in the study. Both men and women are eligible to participate. No specific exclusion criteria apply.

### Participants and recruitment

Four companies are participating in the Health Integrator Intervention study. Two companies with white-collar office employees, including personnel at an insurance company (Länsförsäkringar Alliance) and administrative personnel of Sweden’s largest pharmaceutical retailer (Apoteket AB). We also recruited blue collar employees, i.e. bus drivers, from two companies in Stockholm; at the largest bus transport group in the Nordic countries (Nobina) and at Europe’s largest transport provider (Arriva).

Study participants were recruited in two different ways according to the wishes of each company. The office of human relations at the two companies with white-collar employees sent an inquiry email about the study to their employees. Those who were interested in participating in the study responded to the first email and research personnel then received the email addresses. Bus drivers, whose email addresses are unknown to the employer, were continuously recruited by study personnel on site at the bus garages and asked in person to provide their private email addresses if they are interested in participation. Employees from all four companies that were interested in the study, were thereafter emailed information about the study and a link to access the web-based baseline questionnaire.

Eligible participants were required to give their informed consent for participation in the study in connection with the web-based questionnaire. After an introductory screen displaying information about the study, participants had to consent to participate in order to continue to the questionnaire. At completion of the questionnaire, the respondent was provided with a link to the Health Integrator system.

In the Health Integrator system, each user created an individual user account with personal log in details and responded to a health profile that included 38 questions on physical activity, diet, sleeping habits, stress, alcohol habits, and tobacco use (smoking and oral moist tobacco). The participant also scheduled a time for the baseline meeting with the health coach. The results from the health profile were not visible in the system at this point.

**Fig. 2:**
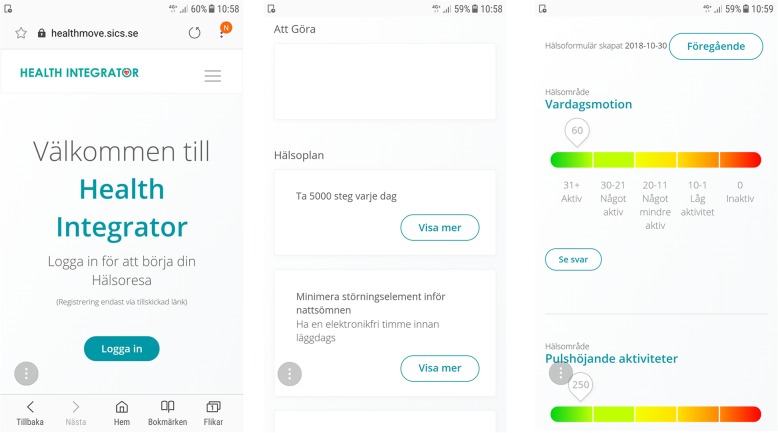
Screen shot from the Health Integrator. To the left, the color green indicates that the reported health habit is very good. In the middle, the color yellow indicates that it is good, while orange and red, found to the right, indicate that the health habit is unhealthy, can be improved, and should be targeted in the intervention

For participants receiving intervention, results from the health profile and the respondent’s status within each behavior were unlocked at the baseline meeting with the health coach and displayed using a colored scale and numbers to indicate if habits were good or could be improved. Green indicated that the reported health habit was very good, yellow that the reported health habit was good, orange that it needed to be improved and red that the reported health habit was associated with health risks and should be improved. See Fig. 2. Results for participants in the control group are unlocked at the follow-up meeting after 3 months.

Written informed consent was additionally obtained at baseline assessments. All study participants meet with study personnel twice: at baseline and at the 3-month follow-up when body composition and blood pressure is assessed, participants respond to the lifestyle questionnaire, and they get a referral for laboratory tests. All participants are also given an accelerometer to measure physical activity during 1 week.

### Randomization and blinding

Once the participant had responded to the baseline questionnaire and scheduled a baseline meeting, they were randomized to one of the three arms using a random allocation list generated with STATA Version 14.0. We randomized by company and in blocks of 6 by gender.

The participant was randomized to 1 of 3 groups: 1) intervention using Health Integrator and a monthly telephone session with the health coach during the 3 months of follow-up (in total two sessions per participant) or 2) intervention using Health Integrator without extra health coach sessions or 3) control group which is not given any lifestyle advise during the intervention period. Subjects in the control group receive access to Health Integrator after 3 months when the active intervention is finished.

Due to the nature of the study, participants are informed about their allocation by study personnel at baseline assessments.

### Intervention

#### Overall

Participants in any of the two intervention groups receive a personalized intervention based on their health profile and tailored to the need of each specific participant. The aim for the individual participant during the intervention is to improve the chosen area of intervention which may be within domains of diet, physical activity, sleeping habits, stress, alcohol, tobacco use or other.

Adverse events or other unintended effects of the trial are voluntarily reported to the health coaches and will be reported.

#### Downloading

The Health Integrator smartphone-application is compatible with both Android (version 4.1 and higher) and iOS (version 8 and higher).

For participants in one of the two intervention groups, each individual’s personal user account in Health Integrator was activated by the health coach at the baseline meeting. Based on the results from the health profile, the participant was helped to navigate to an area mutually chosen by the participant and health coach to be the intervention area of interest.

If, for example, the goal is to increase physical activity, a number of different offers are available to choose from in the Health Integrator system. This could for example be a smartphone-application like Runkeeper or the 7-min-workout, but also conventional training possibilities like a 3-month-training pass at the local gym, or a wrist support band to facilitate rehabilitation. The offers are all free of charge to the participant and quality checked by the health coaches before entered into the Health Integrator system. The participant and the coach have the possibility to choose among 37 offers in total in this version of the system.

#### Goal-setting

The health coach will help participants in the intervention groups to set achievable goals. Once a week the participant records if the weekly goal is met through a goal setting function in the Health Integrator system. This is done with smileys or by checking number of days the goal was met. A reminder is sent out every Sunday at 9.20 pm, prompting the participant to record to what degree the weekly goal was met.

### Study assessments

#### Biomarkers

At the baseline meeting, the participant got a referral to the nearest laboratory for venous blood sample taking. Baseline tests include hemoglobin A1c (HbA1c) in mmol/mol, total cholesterol (mmol/L), Apolipoprotein A1 (g/L) and Apolipoprotein B (g/L). If they are not within normal range at baseline, another measurement is conducted at follow-up, after 3 months, as would be done in clinical practice.

HbA1c is measured using the IFCC (International Federation for Clinical Chemistry and Laboratory Medicine) reference measurement procedure [9], Apolipoprotein A1 and B is analyzed with immunochemistry (turbidimetry) and cholesterol is analyzed with the enzymatic method using photometric reading [10].

#### Body composition and blood pressure

We measure body composition and waist circumference (cm) at baseline and at 3 months of follow-up. Height (cm) is self-reported. Weight is measured to the nearest 0.1 kg. Waist circumference is measured two fingers above the umbilicus to the nearest cm using a non-stretchable Seca 201 circumference measuring tape.

Body composition, including body weight (kg), fat percentage, and skeletal muscle percentage is measured using the body composition monitor OMRON BF 511. By bioelectrical impedance, sending electrical currents from the hands via handheld electrodes to the feet via the scale’s surface electrodes, both the upper and lower body is accounted for in the assessment. An earlier model (OMRON BF 306) has been validated against the criterion method dual-energy X-ray absorptiometry (DEXA) [11].

Blood pressure and pulse is measured with the automatic blood pressure meter OMRON M7 after at least 5 min of rest. We use the upper right arm for measurement, and the participants are instructed to avoid talking or crossing their legs during the procedure.

#### Physical activity measurement

To assess physical activity and sedentary behavior objectively, we use the 3-axial accelerometer ActiGraph wGT3x-BT (www.actigraphcorp.com). The ActiGraph is widely used to capture and record continuous, high resolution physical activity and sleep/wake information. It has been reported to assess energy expenditure due to physical activity, as well as time spent in sedentary behavior, light, moderate and vigorous activity levels, with high accuracy [12, 13]. The ActiGraph is given to the participants at the baseline and at the follow-up meeting. It measures at the frequency of 80 Hz, and is worn at the wrist for 24-h a day during 7 days at baseline and at 3-month follow-up. Participants are also given a log-book to record non-wear time, for example when showering, swimming and bathing. The ActiGraph and the log-book is sent back to the researchers in a prepaid envelope.

#### Questionnaires

*Participant characteristics* are collected with questions on civil status, education, income, use of medications for hypertension, diabetes or hyperlipidemia, estimated number of days on sick leave last year and tobacco use.

*Diabetes risk* is assessed using FINDRISC (Finnish Diabetes Risk Score), a validated prediction tool to identify participants undiagnosed, or at risk of developing diabetes type 2. FINDRISC takes age, body mass index (BMI, kg/m^2^), waist circumference, physical activity, intake of vegetables, fruit and berries, medical treatment of hypertension, history of hyperglycemia and family history of diabetes into account [14].

*Sleep quality and restoration from sleep* is assessed with a short version of the validated Karolinska Sleep Questionnaire [15]. Participants also report habitual sleep as bed time (lights out), rise time and time falling asleep after lights out (subjective sleep latency). Thereby, subjective total sleep can be assessed as the time difference between bed time and rise time, minus sleep latency.

*Dietary intake* is measured using a validated 85 item semi-quantitative food frequency questionnaire (FFQ) [16]. The participants are asked how often they consume different food items, including alcohol, previous months. Response categories varies from seldom/never to three or more times per day. Additionally, five diet questions and two questions about drinking habits developed by the Swedish Board for Health and Social Welfare for use in clinical practice are included [17].

*Eating patterns* are assessed with 21 questions in Three Factor Eating Questionnaire (TFEQ-R21) [18], a widely used and for different populations validated questionnaire. The TFEQ-R21 encompasses three eating behaviors including cognitive restrained eating, emotional eating, and uncontrolled eating.

*Physical activity and inactivity* is assessed with two questions developed for clinical practice, assessing whether or not the participant reaches the guidelines of the World Health Organization for maintaining general health, i.e. ≥150 min of moderate-to-vigorous physical activity per week or ≥ 75 min of vigorous-intensity physical activity per week. Additionally, two questions about sitting time are included. We also ask if the participant has been exercising during the past week. Participants who report to having exercised are asked about the type of activity performed (strength training at the gym, aerobics, swimming/water aerobics, cycling or other) and frequency and duration of performing that activity.

*Health Related Quality of Life*, is assessed with RAND-36, a questionnaire with 36 questions covering eight different dimensions which can be compiled into the two domains: physical component summary scale and mental component summary scale [19].

*Purpose in Life,* i.e. the extent to which a person engages in activities that are personally valued, is measured with a Swedish translation of the 6-item Life Engagement Test [20].

*Perceived stress levels* is assessed using the 14-item Perceived Stress Scale (PSS-14) [21].

*Social support for a healthy lifestyle* (diet, physical activity and less stress) from family, friends and co-workers is assessed with a 6-item questionnaire.

*Stage-of-change, motivation to make lifestyle changes* in terms of diet, physical activity, sleeping habits, stress, alcohol and tobacco is assessed. The response options for these question are in accordance with Prochaskas’ transtheoretical model of behavior change using the five phases of pre-contemplation, contemplation, preparation, action, and maintenance to assess participants’ readiness to change now, within a month, within 6 months, not now, or not at all [22].

#### Sample size and power considerations

The purpose with Health Integrator is to personalize the lifestyle intervention to the particular need of the participant. To estimate the sample size needed to detect a significant difference within the Health Integrator Intervention study, we chose a change in general health score on the RAND 36-Item Health Survey, which includes both physical and mental health, as the primary outcome.

The intervention was planned to include 63 subjects in each group, providing 80% power at the 5% significance level given a two-sided significance level of 5%, to detect a difference in general health score on the RAND 36-Item Health Survey of eight points (55 vs. 63 points) given a standard deviation of 16 [23]. To allow for drop-outs and the fact that the lifestyle intervention is personalized and may focus on different areas of health, we aimed to recruit 300 participants in total.

### Statistical analyses

Descriptive statistics will be produced to describe participant characteristics at baseline and follow-up. Results will be presented by control and intervention groups. Baseline characteristics will be produced to assess the success of the randomization in balancing characteristics. Differences between groups will be assessed using Student’s t-test and chi-square test for continuous and categorical variables, respectively.

Longitudinal data will be analyzed using both generalized estimating equations (GEE) and Mixed Models with a robust covariance structure to assess the effect of both time and the intervention itself on the study outcomes. Any unbalanced baseline characteristics will be controlled for in models. Interactions will be tested for in the models and intention to treat analysis will be performed to account for effects of cross-over and drop-out. Sensitivity analysis to account for missing data will also be done and to further study if the effect of the intervention differs based on participant characteristics.

Finally, we will also analyze how the app is perceived and used. At 3-month follow-up, the intervention group will receive a 16-item questionnaire evaluating the usability and satisfaction with the Health Integrator application. From the Health Integrator system, we will also be able to get descriptive statistics on number of log-ins, fulfilled goals per participant and which lifestyle interventions or services that were the most chosen ones among the participants in the intervention groups.

### Trial status

The study is ongoing. Recruitment of participants started April 16, 2018, when an information letter about the study was sent from the office of Human Relations to potential participants. Randomization and baseline assessments started 1 week later. The outcome assessment at 3-months will continue till December 31, 2018. Follow-up assessment at 6-months will be finalized in March 2019.

## Discussion

WHO has in their global action plan for the prevention and control of non-communicable diseases pinpointed the top four behavioral causes of premature death - physical inactivity, unhealthy diet, tobacco use, and harmful use of alcohol. mHealth has been suggested as one way to take global action [24].

mHealth has the potential to make treatment and prevention widely accessible at a fraction of the current cost. Thousands of mobile apps are available for monitoring of various lifestyle behaviors. However, few are scientifically evaluated and most are stand-alone solutions without the guidance of a professional health coach. A systematic review of the efficacy of interventions that use apps to improve diet and physical activity found that multi-component interventions including for example face-to-face counselling or provision of physical activity equipment in addition to an app, were more successful than a stand-alone app intervention [25]. Based on previous research we developed Health Integrator.

Attractiveness, as well as cost, is of importance to evaluate before scaling up any system. We will evaluate the influence of different levels of guidance by a health coach as adherence has shown to improve when there is a coach involved [26]. However, in-person support in addition to a behavior change intervention using a website was not more successful for weight loss, than only using the website [27]. Thus, the benefit of adding a health coach is uncertain.

Attrition is common in mHealth interventions despite the fact that participation is independent of time and place, and thus very flexible. Reminders have been shown to decrease attrition in clinical studies, also when they are automatic [28]. The Health Integrator system comprise a number of different lifestyle interventions, but independent of area for lifestyle improvement, a weekly reminder in the form of an evaluation of the goal fulfillment of the week is being sent out. Goal setting as such, is also an important behavior change technique [29].

Strengths of the study include that it is a randomized controlled trial in a relatively large sample, including both blue- and white-collar employees. Interventions at the workplace can be an effective health promotion strategy, which has also been endorsed by the World Health Organization which stated that, “the workplace directly influences the physical, mental, economic and social well-being of workers and in turn the health of their families, communities and society. It offers an ideal setting and infrastructure to support the promotion of health of a large audience”. Our outcomes are measured objectively (accelerometer measured physical activity, anthropometric measures, blood pressure and laboratory values) and we use a number of validated questionnaires. Further, the Health Integrator app is available for both Android and iOS devises, which increases the likelihood that most smartphone users can download it.

Limitations include that we only recruit employed individuals. This may lead to a selection of healthier people than the general population, and in this type of study, potentially to a smaller effect of the actual intervention. Another limitation may be that a smartphone is a prerequisite for inclusion to the trial. However, smartphone use is widespread in Sweden. In 2017, among those aged 26–35, 99% owned a smartphone, while this figure decreased slightly to 83% at the age of retirement [30].

For technical reasons, we were not able to order the laboratory tests through the Health Integrator system. This is a planned feature in future versions. Instead, this is done manually and the participant has to bring a paper-based referral to the lab for blood sample taking. Results from analysis are thereafter sent from the lab to the study physician via regular mail. This means that the health coach has the results of the laboratory tests one or 2 weeks into the intervention. For future dissemination of Health Integrator, ordering laboratory tests digitally directly in the platform and receiving the results electronically before the baseline meeting has several benefits. This would decrease the work load, the risk of human error, lost letters, make better use of the potential of mHealth, but above all, pathological results such as hyperlipidemia or hyperglycemia can be targeted with lifestyle intervention, which should be initiated already at baseline.

The Health Integrator system is a novel approach to offer personalized and quality checked health interventions in various lifestyle areas, using a mixture of mHealth and conventional interventions strategies. The system is built to be flexible and can easily be adapted based on the findings of this study. If this solution proves to be useful, it can be transferred to be used in various settings, for example as an occupational benefit.

## Abbreviations

App: Application
EIT: European Institute of Innovation and Technology
FFQ: Food frequency questionnaire
GEE: Generalized estimating equation
HbA1c: Hemoglobin A1c
IFCC: International Federation for Clinical Chemistry and Laboratory Medicine
mHealth: Mobile health
TFEQ-R21: Three Factor Eating Questionnaire Revised-21 items
WHO: World Health Organization
